# A systematic review and meta-analysis of the genetic characterization of human echinococcosis in Iran, an endemic country

**DOI:** 10.4178/epih.e2019024

**Published:** 2019-06-15

**Authors:** Abolghasem Siyadatpanah, Davood Anvari, Amir Emami Zeydi, Seyed Abdollah Hosseini, Ahmad Daryani, Shahabeddin Sarvi, Christine M. Budke, Reza Esmaeelzadeh Dizaji, Mohammad Ali Mohaghegh, Mohammad Hasan Kohansal, Samira Dodangeh, Reza Saberi, Shirzad Gholami

**Affiliations:** 1 Ferdows Paramedical School, Birjand University of Medical Sciences, Birjand, Iran; 2 Infectious Diseases Research Center, Birjand University of Medical Sciences, Birjand, Iran; 3 Department of Parasitology, Student Research Committee, Toxoplasmosis Research Center, Mazandaran University of Medical Science, Sari, Iran; 4 Department of Microbiology and Immunology, School of Medicine, Iranshahr University of Medical Sciences, Iranshahr, Iran; 5 Department of Medical-Surgical Nursing, Nasibeh School of Nursing and Midwifery, Mazandaran University of Medical Sciences, Sari, Iran; 6 Department of Parasitology, Toxoplasmosis Research Center, Mazandaran University of Medical Sciences, Sari, Iran; 7 Department of Veterinary Integrative Biosciences, College of Veterinary Medicine Biomedical Sciences, Texas A&M University, College Station, TX , USA; 8 Department of Poultry Disease, Faculty of Veterinary Medicine, University of Tehran, Tehran, Iran; 9 Health Sciences Research Center, Department of Laboratory Sciences, School of Paramedical Sciences, Torbat Heydariyeh University of Medical Sciences, Torbat Heydariyeh, Iran; 10 School of Medicine, Bam University of Medical Sciences, Bam, Iran

**Keywords:** Cystic echinococcosis, Genotype, Human, Systematic review, Meta-analysis, Iran

## Abstract

Human echinococcosis is an infectious disease caused by tapeworms belonging to the species *Echinococcus*. This parasite has a worldwide distribution and is considered a neglected tropical disease by the World Health Organization. Due to the diversity of *Echinococcus* spp. hosts, as well as variation in geographical, climatic, and socio-ethnic conditions, the question of the strains or genotypes of *Echinococcus* spp. that are involved in human infections is important. The aim of this study was to provide a summary of the available data on genotypes of *Echinococcus* obtained from the Iranian population. Four international databases (PubMed, Scopus, Science Direct, and Web of Science) and 4 Persian databases (Magiran, Scientific Information Database, Iran Medex, and IranDoc) were searched for cross-sectional studies that reported the genotypes of *Echinococcus* spp. in human echinococcosis cases using molecular methods in Iran through July 2018. The Newcastle-Ottawa Scale was used to assess the quality of the selected studies. A total of 559 cases of human cystic echinococcosis were reported in the 21 included articles. The majority of cases belonged to genotype G1 (89.2%; 95% confidence interval [CI], 80.1 to 95.8), genotype G6 (8.2%; 95% CI, 2.8 to 15.9), and genotype G3 (2.3%; 95% CI, 1.1 to 3.9). Since genotype G1 of *Echinococcus* appears to be the most prevalent genotype affecting humans in Iran, disease control initiatives aimed at sheep intermediate hosts may be the most beneficial. In addition, educational programs and serological screening in individuals may help reduce the national impact of the disease.

## INTRODUCTION

Human echinococcosis is an infectious disease caused by tapeworms belonging to the species *Echinococcus*. This parasitic zoonosis has a worldwide distribution and is considered a neglected tropical disease by the World Health Organization [1,2]. Adult worms live in the small intestine of carnivores (for example, dogs, jackals, foxes, and wolves) and the eggs are excreted through the feces of these animals. *Echinococcus* eggs are ingested by intermediate hosts, including ruminants, camels, horses, and pigs, where the parasite forms metacestode cysts [3,4]. Humans can also act as aberrant intermediate hosts through direct infection (via direct contact with an infected definitive host) or indirect infection (via ingesting contaminated food or water) [4,5].

The 2 major species of medical and public health importance are *Echinococcus granulosus* and *Echinococcus multilocularis*, which cause cystic echinococcosis (CE) and alveolar echinococcosis, respectively. Both are serious and severe diseases, the latter especially so, with high case fatality rates and a poor prognosis if managed incorrectly. Based on phylogenetic studies, *E. granulosus* sensu lato consists of 10 genotypes or group complexes, which include *E. granulosus* sensu stricto (genotypes G1-G3), *Echinococcus equinus* (genotype G4), *Echinococcus ortleppi* (genotype G5), *Echinococcus canadensis* (genotypes G6-G8, G10), and *Echinococcus felidis* (lion strain). All of these genotypes, except genotype G4, have been shown to cause human disease [6,7]. CE can affect any part of the body, but the liver and lungs are the most commonly affected organs. Clinical symptoms vary depending on the cyst’s size and anatomical location, with many patients remaining asymptomatic for years or never developing symptoms [8]. In addition to its health consequences, CE also has an economic impact on affected communities and regions. The estimated global economic loss due to human infections was estimated to be US$200-800 million, annually [9].

CE is endemic in Iran and the reported prevalence of *Echinococcus* spp. infection in Iranian dogs ranges from 5% to 49%. The prevalence of CE in Iranian sheep and camels has been reported to be as high as 88% and 64%, respectively [10]. The frequency of CE detected in surgical cases ranges from 1.1 to 18.3 per 100,000 population depending on region [2,10]. Molecular identification of locally circulating *Echinococcus* genotypes is important for gaining insights into parasite prevalence and disease ecology in the various regions. Molecular identification also plays an important role in the development of control strategies, including the identification of locally important definitive and intermediate hosts [8, 11-14]. Numerous studies have been performed on genotyping of *E. granulosus* from Iran. However, no study has systematically analyzed this information. The aim of this systematic review was to provide a summary of the available data on *Echinococcus* genotypes obtained from the Iranian population.

## MATERIALS AND METHODS

### Search strategy

Four international databases (PubMed, Scopus, Science Direct and Web of Science) and 4 Persian databases (Magiran, Scientific Information Database, Iran Medex, and IranDoc) were searched for articles (full text) published online in the English and Persian languages from January 1999 to July 2018. The keywords, used alone or in combination, were “cystic echinococcosis,” “*Echinococcus granulosus*,” “hydatid cyst,” “hydatid disease,” “hydatidosis,” “hydatidoses,” “genotype,” “genetic variation,” and “Iran”. In addition, to avoid missing any articles, the references of the identified articles were carefully checked.

**Figure 1.**
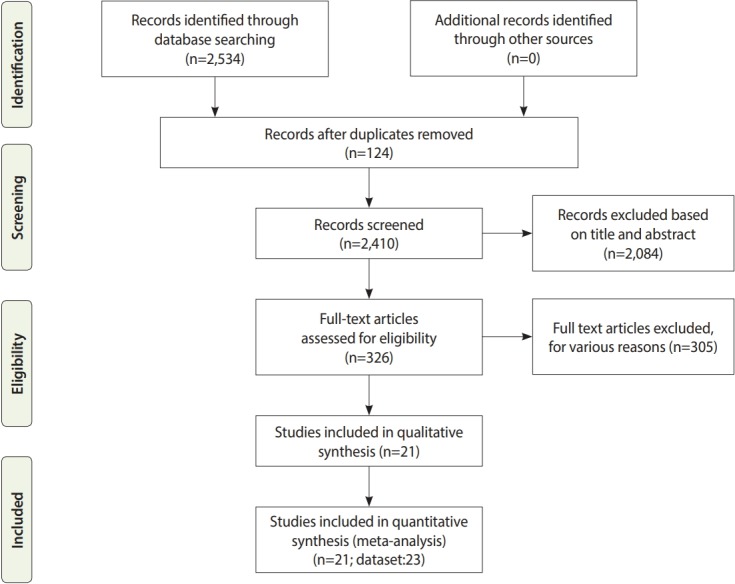
Flow diagram of the systematic review and meta-analysis process.

The literature search identified 2,534 papers, of which 124 were excluded as duplicates. After a primary screening of the titles and abstracts, an additional 2,084 studies were excluded. After reading the full text of the remaining articles, 305 other papers were eliminated. Four studies were added after reviewing the references of the identified papers. Finally, 21 eligible studies were identified for inclusion in the systematic review (Figure 1 and Table 1) [15-35].

### Inclusion and exclusion criteria

A paper was included if it fulfilled the following criteria: a crosssectional study that investigated human echinococcosis by any molecular diagnostic method that detected genotypes of *Echinococcus* spp. in Iran. Duplicate manuscripts, case reports or case series, letters to the editor, review articles, animal studies, non-molecular studies, and articles with insufficient information were excluded.

### Data extraction

After completing the search, the chosen articles were independently assessed by 2 authors (AS & SG). All duplicate and irrelevant articles were excluded after evaluating their titles, abstracts, and full texts. Any disagreements between the 2 authors were resolved by consultation with another author (DA). The characteristics of each manuscript were extracted using a predesigned datacollection spreadsheet. The recorded information included the first author, year of publication, study location, study design, number of total collected samples, and number of samples for each identified genotype.

### Quality assessment

In the current study, the Newcastle-Ottawa Scale was utilized to assess the quality of cross-sectional studies (low quality, < 3.50; moderate quality, 3.60-5.25; and high quality, 5.26-7.00) [36]. Only articles with at least an acceptable quality (> 3.50) were included in the meta-analysis.

### Statistical analysis

The meta-analysis was performed using StatsDirect (https://www.statsdirect.com/). The standard error for each study estimate was calculated from a binomial distribution formula. We pooled each study estimate using a random-effects model to obtain an overall summary estimate of each *Echinococcus* isolate genotype (G1, G2, G3, and G6), as well as the accompanying 95% confidence intervals (CIs). Heterogeneity between the results was assessed based on the Q test and the I^2^ indicator [37-39]. In addition, the Egger test was used to investigate the presence of publication bias.

### Ethics statement

This study is a systematic review and does not deal with human participants.

## RESULTS

Data were available from 15 Iranian provinces. The included studies demonstrated wide variation in the proportion of genotypes identified in individual studies.

**Figure 2.**
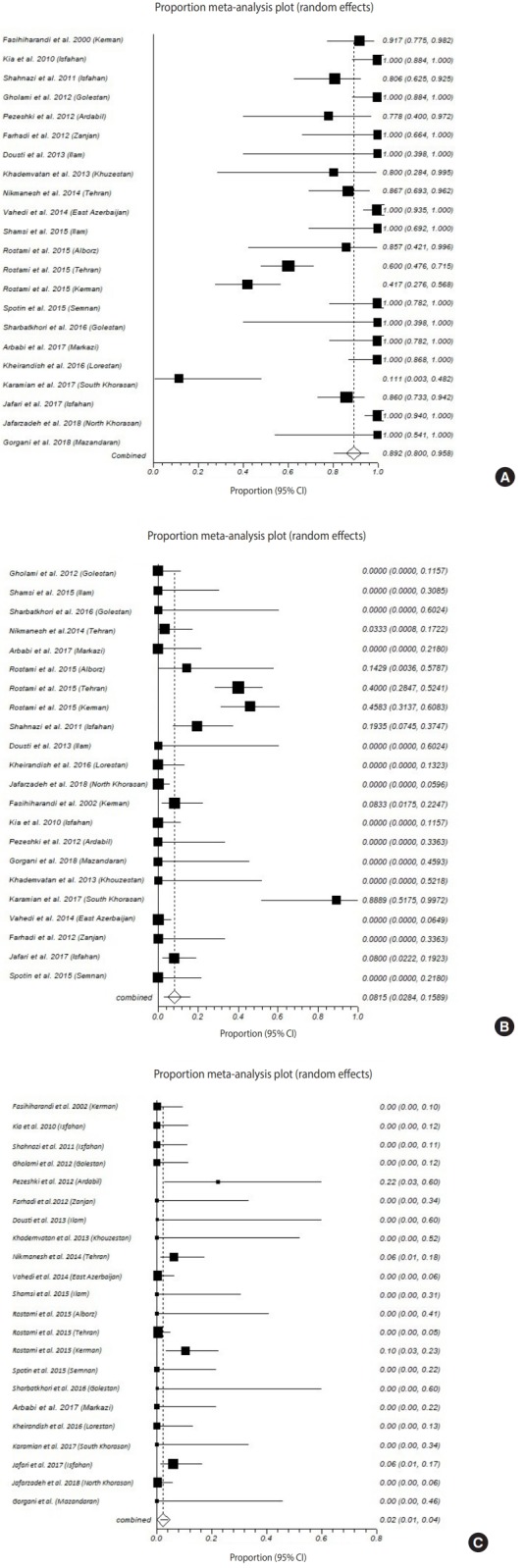
Forest plot of the proportion of cystic echinococcosis cases due to Echinococcus granulosus genotypes (A) G1, (B) G6, and (C) G3 in Iran. CI, confidence interval.

A total of 559 cases of human CE were reported in the 21 included studies. The majority of cases belonged to genotype G1 (89.2%; 95% CI, 80.1 to 95.8), genotype G6 (8.2%; 95% CI, 2.8 to 15.9), and genotype G3 (2.3%; 95% CI, 1.1 to 3.9), respectively (Figure 2 and Table 2). Since only a single CE case attributed to the genotype G2 has been identified in Iran, this genotype was not included in the meta-analysis (Table 1). The Egger regression test indicated that publication bias was significant for genotypes G1 and G6, but not for genotype G3 (Table 2).

## DISCUSSION

CE is an emerging neglected disease and a public health concern in the Middle East that results in substantial economic losses [40,41]. Molecular surveys are considered to be the gold standard for the differential diagnosis of human echinococcosis. This method is the only way to identify the species of *Echinococcus* (genotypes). In contrast to the widespread use of serological and imaging tests, molecular assays are rarely used for clinical diagnoses, perhaps due to the delay in diagnosis. Making a correct diagnosis is an essential prerequisite for the effective treatment of this disease, as misdiagnosis or unclear diagnosis could eventually lead to relapse or metastasis, even after surgery. The genetic characterization of *E. granulosus* using polymerase chain reaction–based methods has yielded a deeper understanding of its global epidemiology [42]. At present, 4 *E. granulosus* genotypes (G1, G2, G3, and G6) have been identified in human CE patients in Iran [43]. Similar to our study, a systematic review of human cases worldwide showed that 72.9% were caused by *E. granulosus* genotypes (G1), 12.2% by G6, 9.6% by G7, 3.2% by G3, 1.0% by G5, 0.8% by G2, 0.2% by G10, and 0.1% by G8 [44].

Worldwide, the G1 genotype is responsible for the majority of human CE cases [42,44], and this genotype is largely maintained in a domestic sheep/dog life cycle. Cysts found in older sheep are often fertile [45,46]. While human cases from Pakistan and Afghanistan have largely involved the G1 genotype, more diverse CE genotypes have been isolated from human subjects in Turkey and Iraq [47].

In the present study, genotype G6 (camel strain) accounted for 8.15% of isolates collected from Golestan, Tehran, Alborz, Kerman, Isfahan, Khuzestan, South Khorasan, and Semnan Provinces. Due to the special weather conditions (heat and aridity) required for breeding camels in Iran, this animal is only raised in a few provinces, including Khorasan Razavi, South Khorasan, Semnan, Sistan and Baluchestan, and Yazd Provinces. For this reason, this genotype has not been observed in all provinces of Iran [48]. Genotype G6 appears to be the second most prevalent *E. granulosus* genotype in Iran, which is comparable to recent information from South American countries [44]. This genotype has been reported in a variety of countries, including Brazil, Kenya, Turkey, China, and southeastern Romania [49].

The results of this systematic review and meta-analysis show that genotype G3 (buffalo strain) was found in 4 provinces of Iran, including Isfahan, Ardabil, Kerman, and Tehran Provinces. This genotype has also been reported in humans in Italy, Romania, Turkey, India, Tunisia, and Brazil [47]. This genotype has also been reported in sheep, goat, cattle, and pigs in Pakistan, Italy, and Greece [50,51].

The G2 strain was found in 1 case from Kerman Province [20]. This is not unexpected, since one of the main intermediate hosts of this genotype (Tasmanian sheep) are not found in Iran. However, in 2012, Parsa et al. [52] identified this genotype, for the first time, from a dog in Iran. The absence of genotype G7 (pig strain) in human cases in Iran may be partially ascribed to the lack of pig breeding in Islamic countries.

This systematic review has the following limitations: (1) none of the included studies evaluated variables such as patients’ age, sex, and type of immunity, (2) the studies analyzed herein contained insufficient information about patients’ condition or the severity of the disease, and (3) the included studies had no uniform sample size. These limitations may have significant implications for our understanding of the epidemiological aspects of this infection among humans in Iran. In addition, many questions remain to be answered in future investigations.

## CONCLUSION

Since genotype G1 appears to be the most prevalent genotype affecting the Iranian population, disease control initiatives aimed at sheep intermediate hosts may be the most beneficial. Moreover, educational programs and serological screening in the population may help to reduce the national impact of human echinococcosis. Further studies are needed to describe the comprehensive epidemiology of this disease at a national level in Iran.

## Figures and Tables

**Table 1.** Basic characteristics were identified in the studies^1^

Study	Province	Genotypes	Human CE (n)	QA
Harandi et al., 2002 [25]	Kerman	G1, G6	36	7
Kia et al., 2010 [26]	Isfahan	G1	30	7
Shahnazi et al., 2011 [21]	Isfahan	G1, G6	31	7
Gholami et al., 2012 [15]	Golestan	G1, G6	30	6
Sadjjadi et al., 2013 [28]	Khuzestan	G6	3	6
Pezeshki et al., 2013 [27]	Ardabil	G1, G3	11	7
Dousti et al., 2013 [22]	Ilam	G1	4	7
Khademvatan et al., 2013 [30]	Khuzestan	G1	5	6
Vahedi et al., 2014 [32]	East Azerbaijan	G1	55	7
Nikmanesh et al., 2014 [18]	Tehran	G1, G3, G6	47	6
Rostami et al., 2015 [20]	Alborz	G1, G6	7	7
Rostami et al., 2015 [20]	Tehran	G1, G6	70	7
Rostami et al., 2015 [20]	Kerman	G1, G2, G3, G6	48	6
Spotin et al., 2015 [35]	Semnan	G1, G6	7	7
Farhadi et al., 2015 [33]	Zanjan	G1	9	7
Shamsi et al., 2015 [16]	Ilam	G1	10	7
Sharbatkhori et al., 2016 [17]	Golestan	G1	4	7
Kheirandish et al., 2017 [23]	Lorestan	G1, G3	26	7
Karamian et al., 2017 [31]	South Khorasan	G1, G6	9	7
Arbabi et al., 2017 [19]	Markazi	G1	15	7
Jafarzadeh et al., 2018 [24]	North Khorasan	G1	60	7
Gorgani-Firouzjaee et al., 2018 [29]	Mazandaran	G1	6	7
Jafari et al., 2018 [34]	Isfahan	G1, G3, G6	50	6

CE, cystic echinococcosis; QA, quality assessment.

1 Type of studies were cross-sectional study.

**Table 2.** Prevalence, publication bias, and heterogeneity of genotypes of human echinococcosis in Iran

Genotype	Prevalence, % (95% CI)	Cochran Q	df	I² (%)	p-value	Egger bias	p-value
G1	89.2 (80.1, 95.8)	188.9	21	88.9	<0.001	-2.3	0.011
G6	8.2 (2.8, 15.9)	164.9	21	87.3	<0.001	-1.4	0.025
G3	2.3 (1.1, 3.9)	26.8	21	21.6	0.178	0.4	0.086

CI, confidence interval; df, degree of freedom.
